# Understanding the needs of undergraduate healthcare students in relation to suicide prevention training: A qualitative study

**DOI:** 10.1371/journal.pone.0327538

**Published:** 2025-07-09

**Authors:** Kerrie Gallagher, Ellen Walls, Clíodhna O’Brien, Eve Griffin, Grace Phillips, Michelle O’Driscoll

**Affiliations:** 1 National Suicide Research Foundation, Cork, Ireland; 2 School of Public Health, University College Cork, Cork, Ireland; 3 School of Pharmacy, University College Cork, Cork, Ireland; Universidad Pública de Navarra, SPAIN

## Abstract

**Background:**

International evidence indicates that many healthcare workers are not adequately prepared to support patients during suicidal crises. As a result, healthcare students need appropriate training in order to effectively intervene once they qualify. In Ireland, there is no standardised suicide prevention training for students who are undertaking a degree in the health professions. The aim of this study was to explore the perspectives of undergraduate healthcare students to inform the design, implementation and evaluation of a suicide prevention module.

**Methods:**

Ethical approval was obtained to conduct focus groups with undergraduate healthcare students. Recruitment was by convenience and snowball sampling within the research team’s networks. Eligible participants for this study were students over the age of 18 who were enrolled in an undergraduate healthcare course. Focus groups were held online via MS Teams, and were subsequently transcribed and qualitatively analysed using Braun and Clarke’s thematic analysis.

**Results:**

A total of 12 participants from Graduate Entry Medicine (*n* = 9) and Pharmacy (*n* = 3) took part across four focus groups, which averaged 53 minutes in duration. Six key themes emerged from the data: (i) the need for suicide prevention training for healthcare students; (ii) *“tiptoeing”* around the topic of suicide; (iii) creating a safe environment; (iv) student self-care and support – different strokes for different folks; (v) module implementation and (vi) appropriate teaching methodologies.

**Conclusions:**

Healthcare students would positively receive the embedding of suicide prevention training into their respective degrees. The findings of this study serve to include the student voice in a suicide prevention curriculum design, and have since been used to inform its subsequent pilot and evaluation. This will ultimately improve the suicide prevention knowledge and competence of our future healthcare professionals.

## Introduction

It is widely recognised that suicide is a serious public health issue. More than 700,000 deaths are attributed to suicide each year, and it is the leading cause of death among individuals between the ages of 15 and 29 [1]. Recent research from Ireland has shown that more than 80% of suicide decedents had contact with a healthcare provider in the year prior to their death [2]. These findings highlight the important role that future healthcare professionals could potentially play in suicide prevention efforts.

Suicide prevention is a complex challenge that requires a coordinated, multidisciplinary approach at all levels of the healthcare system. In community settings, healthcare professionals (HCPs) support individuals in distress who are not in immediate danger by referring them to appropriate services such as crisis helplines or community-based mental health supports. Additionally where somebody is deemed to be a risk to self or others, HCPs are expected to facilitate access to emergency services, where clinical HCPs provide additional, more specialised care [3]. These referral pathways form an integral part of suicide prevention systems, allowing for timely and appropriate intervention [4].

However, despite these systems and referral pathways being in place, international research indicates that HCPs—including those in training—often lack the knowledge, skills, and confidence needed to identify suicide risk, intervene effectively, or understand the referral options available to [5] them. Significant gaps in mental health education persist across healthcare curricula [6]. Stigma or negative attitudes amongst healthcare professionals can further hinder care by delaying help-seeking, reducing treatment adherence, and compromising patient safety and quality of care [4,7,8].

This highlights the need to integrate suicide prevention training into the current curricula of third level health and social care degree courses. Equipping future healthcare professionals with the skills to engage compassionately and confidently with individuals in crisis and to navigate appropriate support systems is crucial. Evidence has shown that such training can improve knowledge and attitudes toward suicide, and increase students’ self-perceived ability to assist someone at risk [9–11]. These benefits have been documented across healthcare and community contexts, particularly when education is formally integrated into third-level programmes [11–13]. However despite this extensive evidence, there is significant variability in suicide prevention education competencies, learning outcomes and delivery methods for this cohort internationally [14,15].

In the Irish context, there is currently no standardised approach to suicide prevention education across undergraduate health and social care programmes. While some higher education institutions do cover aspects of mental-health or suicide-related content, delivery is inconsistent and often dependent on individual educators or available resources. One of the key challenges in embedding suicide prevention education more systematically is the already overcrowded nature of healthcare curricula. Students are required to develop a broad range of clinical skills in a limited timeframe, and suicide prevention education must often compete with other training priorities. In recognition of this educational gap, and to respond appropriately, the Irish Health Service Executive, National Office for Suicide Prevention (HSE-NOSP) commissioned researchers at the National Suicide Research Foundation (NSRF) to develop a national, standardised undergraduate suicide prevention curriculum for health and social care students. This aligns with Action 5.4.4 of *Connecting for Life: Ireland’s National Strategy to Reduce Suicide* [16]. Initial work included a scoping review of the literature [17], a national survey of healthcare academics, and the establishment of an academic consultation group which confirmed the need for standardised training in this area. This led to the design of an initial draft of the proposed module content for further consideration by healthcare students.

The aim of this qualitative study was to include the perspectives of undergraduate healthcare students in the design, delivery, and implementation of this module prior to its pilot and evaluation.

## Materials and methods

This study was granted ethical approval by the Social Research Ethics Committee in University College Cork (log number 2023−158).

### Recruitment and participants

Participants were initially recruited through convenience sampling from the research team’s networks within their academic institution in August 2023. This involved approaching gatekeepers in the respective Schools to request the distribution of a recruitment email to all 2^nd^, 3^rd^, and 4^th^ year students in undergraduate healthcare courses. The team also contacted various student societies related to health and social care to act as gatekeepers, and posted on social media pages that would reach the intended cohort. Those who agreed to take part were invited to participate in snowball sampling, extending the invitation to friends that may also be interested in contributing to the research.

Inclusion criteria were students over the age of 18 who were enrolled at the time of recruitment in an undergraduate healthcare course. Recruitment focused on the researchers’ own institution, but ethical approval was also obtained to expand the scope beyond this to other universities via social media if needed.

Recruitment for the study was continued until it was determined that the final focus group presented no new emerging themes or sub-themes. It was also ensured that there was appropriate representation from participants in the later years of their undergraduate study, who would be best placed to advise on the embedding of the module into curricula. Although the recruitment period of two weeks was limited by the time-frame in which the intern researcher was available, the researchers were satisfied that the research question had been explored and answered in sufficient depth.

Students who were interested in taking part in the focus groups and contacted the research team for further information were provided with a participant information leaflet and a secure electronic Qualtrics link to provide informed consent and choose a focus group time that was convenient for them. Participants were not remunerated for their contributions. They were informed that participation would not affect their grades, or the service they would receive from the institution in which they were recruited from.

Prior to each session, participants were sent a document outlining the proposed module content, including the core topics, learning outcomes and associated learning activities (S1 File. Suicide Prevention Module Content Information). The purpose of this was to inform the discussion and facilitate a more in-depth reflection on module content, and delivery considerations.

### Data collection

Focus groups were held on Microsoft Teams using a password-protected private joining link that was sent to participants. Further security measures included having a virtual ‘waiting area’ where participants had to be admitted by researchers before they could join the conversation. Each focus group was run by two researchers – a lead facilitator who carried out the session and guided discussion, and a co-facilitator who was there in a supportive capacity. The co-facilitator monitored the discussion, took field notes, and was also there to deal with any unexpected interruptions in a safe and timely manner. A safety protocol was put in place in the event of a security breach. The discussion was guided by a topic guide which provided additional prompts for the researchers to utilise if needed (S2 File: Topic Guide). Each focus group began with brief introductions to ascertain participants’ course, year of study, and any previous suicide prevention education experience. Subsequent questions explored participants’ opinions of the proposed module content, student needs and supports, teaching methodologies and future recommendations.

### Ethical considerations

Though it was not envisaged that this research would be distressing, due to the focus on training development and implementation rather than lived experience, suicide can be an emotive and distressing topic nonetheless. To safeguard the participants, a detailed safety protocol was put in place . This included signposting the participants to appropriate resources at the beginning and end of the sessions, having the co-facilitator there in a supportive role, and flagging the research team’s email addresses so that participants could reach out at any time during or after the session if needed. 

Prior to recording and transcribing the session on Microsoft Teams, participants were reminded that they had consented to audio and video recording. They were informed that they could withdraw their participation at any time during the focus group, and if they wanted to leave the session that researchers would contact them to ensure that they were not distressed. They could also withdraw their contributions to the research within 24 hours of the session by emailing a member of the research team. Data would be combined and no longer identifiable beyond this time-frame. Interview results were not sent back to the participants, although they were entitled to request a copy of their transcript or the study findings if they wished.

### Analysis

Microsoft Team transcripts were checked for discrepancies by a member of the research team to ensure accuracy, and all participants’ data were then pseudonymised. Transcripts were processed on password-protected encrypted laptops using a secure server. All video recordings were then deleted to ensure participant confidentiality.

Transcripts were analysed using Braun and Clarke’s thematic analysis [18]. By using this method, codes and themes can be generated from qualitative data in a systematic and accessible manner [18,19], and themes are allowed to be identified naturally, rather than constraining the data to prerequisite categories. The six steps used in the analysis were as follows: (i) familiarisation of data – all researchers read the transcripts to immerse themselves in the findings; (ii) generation of codes – one researcher generated initial codes for all focus group transcripts, and a second researcher re-coded one focus group to confirm agreement in approach; (iii) generation of themes – one researcher generated themes by grouping of the codes using Microsoft Excel and a tabulated version of the transcript, and these initial themes and the codes comprising them were discussed amongst the research team; (iv) reviewing themes – themes were checked for consistency and accuracy through discussion by the research team; (v) defining and naming of themes – the key themes and sub-themes were named and finalised; (vi) reporting of findings – a narrative to describe and explore the themes and sub-themes was created [19].

Credibility was ensured by prolonged immersion in the data by the researchers. It was acknowledged that as the intern researcher was a graduate entry medical student and the senior researcher was a pharmacist, they could potentially bring their own biases to the research. Furthermore, triangulation was facilitated by comparing the findings to those of an in-depth scoping review of the literature.

Dependability was ensured by documenting the research process throughout, including all decisions taken in relation to study design, recruitment and data analysis. Transferability was addressed by describing the context and sampling strategy in detail, and confirmability was ensured by regular in-depth discussions amongst the research team to confirm interpretations and findings.

## Results

Twelve participants took part in the study across four focus groups. Within the sample, seventy-five percent (*n* = 9) were medical students, and the remainder were pharmacy students (*n* = 3). Half of the participants identified as male, and half as female. Seventy-five percent of participants indicated that they had no previous experience of suicide prevention training. Participant demographics are presented in Table 1. Focus groups averaged 53 minutes in duration (range 45.4mins – 61.1mins).

**Table 1:** Participant Demographics.

Participant code	Gender	Degree	Year	Previous experience of suicide prevention training
PA	M	Medicine	3^rd^	N
PB	F	Medicine	3^rd^	N
PC	M	Medicine	3^rd^	N
PD	F	Medicine	3^rd^	Y
PE	F	Medicine	3^rd^	N
PF	F	Medicine	3^rd^	N
PG	M	Medicine	3^rd^	N
PH	F	Medicine	3^rd^	Y
PI	M	Medicine	3^rd^	Y
PJ	F	Pharmacy	4^th^	N
PK	M	Pharmacy	4^th^	N
PL	M	Pharmacy	4^th^	N

**Fig 1:**
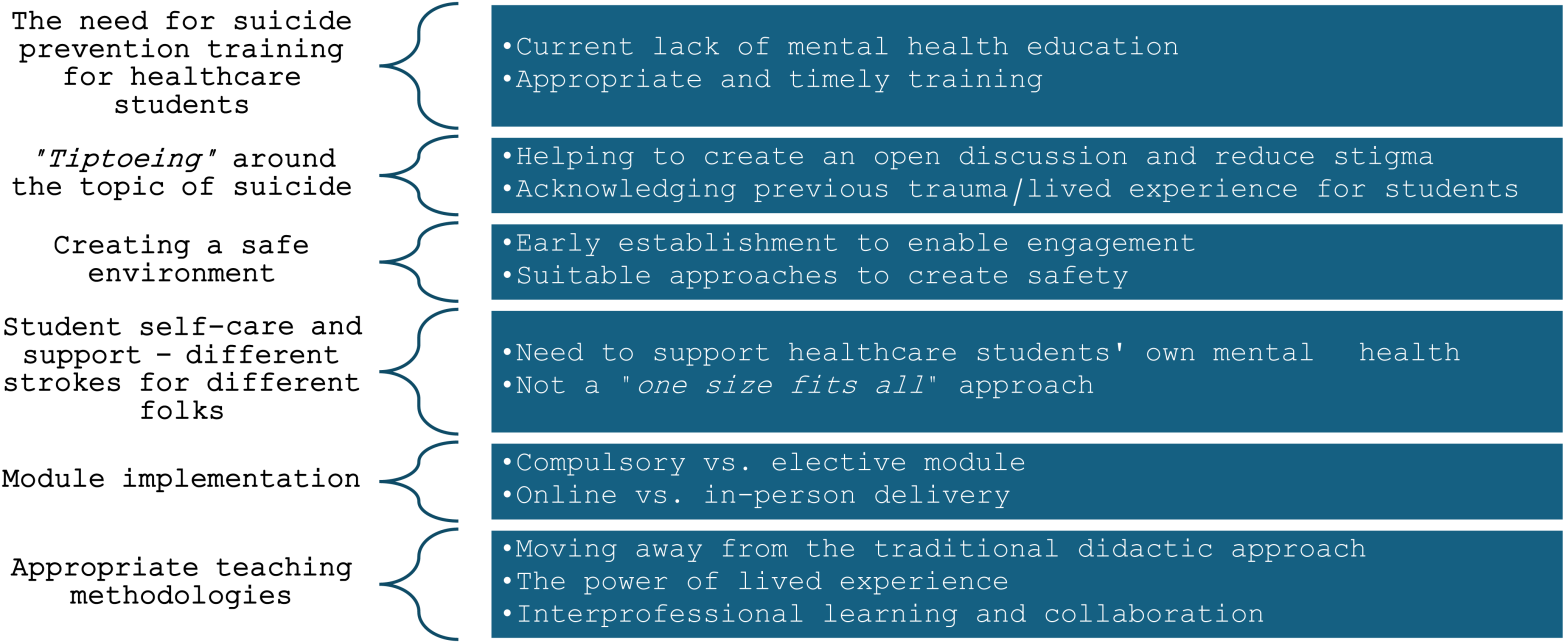
Themes and subthemes.

In total, six main themes emerged from the thematic analysis: (i) the need for suicide prevention training for healthcare students; (ii) *“tiptoeing”* around the topic of suicide; (iii) creating a safe environment; (iv) student self-care and support – different strokes for different folks; (v) module implementation and (vi) appropriate teaching methodologies (see Fig 1).

### Theme 1: The need for suicide prevention training for healthcare students

The need for suicide prevention training for healthcare students emerged strongly from the focus groups, and was echoed by all participants. The relevance of such education to their future healthcare professions was unanimously recognised, and the lack of existing training was highlighted. The two sub-themes that emerged within this theme were (i) current lack of mental health education, and (ii) appropriate and timely training.

#### (i) Current lack of mental health education.

Focus group findings confirmed what previous research had found, which was that despite suicide prevention training being deemed “*extraordinarily important*” (PD), both medical and pharmacy participants felt that there was a lack of focus on mental health teaching in their respective degree curricula. When mental health or suicide was addressed in their degree teaching, participants felt it was more focused on pathophysiology or pharmacology, rather than on how to act to support suicidal patients. The content that was covered as part of a psychiatry placement for medical students was not considered sufficient to meet the needs of a future healthcare professional, and it was felt that it should be an integral, standard part of their healthcare course.

*“Suicide is something that was not highlighted to us, like nearly enough. I honestly… I’m trying to think if it has ever been.”* (PB)

#### (ii) Appropriate and timely training.

The general sentiments of participants were that despite this being a subject which they did not have much experience with, they felt it was fundamental for them to be taught it as future healthcare professionals. Participants felt that by having specific training in suicide prevention, it would help them to gauge how to react correctly in their professional career when faced with someone who has suicidal ideation.

*“Bringing in a module like this is a massive step forward in terms of preparing healthcare professionals for going out into the world when they qualify. It would be a welcome change.”* (PL)

### Theme 2: “Tiptoeing” around the topic of suicide.

The focus groups highlighted some potential trepidation and conservative attitudes towards the topic of suicide that may be encountered in the delivery of the module, which they felt would be reflective of society’s general attitude towards suicide as an issue. The proposed reasons for this included stigma, and the personal experiences of participants. The sub-themes that emerged fhere were (i) help to create an open discussion and reduce stigma, and (ii) acknowledging previous trauma/lived experience for students.

#### (i) Help to create an open discussion and reduce stigma.

Students expressed the importance of having this training in their undergraduate curricula, as it would help to increase awareness of suicide and mental health, thus helping to alleviate the often prevalent stigma associated with such conversations. Normalising the topic of suicide would only help in breaking down the barriers and opening up the discussion going forward to enable suicide prevention for patients. The environment for these conversations needed to be safe and secure, making the dialogue easier for students to engage with and contribute to.

*“I think one of the biggest barriers to this sort of training is it’s such a topic that people want to kind of tiptoe around or they don’t want to bring up, and they don’t want to upset people. But like, it definitely does need to be normalised more, because I think that would make people a lot more forward about feelings that they may be having.”* (PF)

*“By having people actually talk about these kinds of things openly, it creates that environment that you’re kind of aiming for, that safe environment where it becomes easier and easier for people to discuss it, and it becomes less stigmatised.”* (PD)

#### (ii) Acknowledging previous trauma/lived experience for students.

In addition, participants acknowledged the difficulty that students with lived experience of suicide may encounter while exploring this topic. They may have been bereaved by family or friends’ suicides. Furthermore, given that participants are part of the at-risk cohort of university students, and soon to be part of the at-risk cohort of healthcare professionals, the chance of personal lived experience featuring amongst participants is also high. This could lead to displays of emotion, distress, or difficulty engaging with the content. Participants acknowledged that awareness of what could be in the room, and the need for sensitive content delivery was vital, with appropriate preparation and training to support same.

“*I suppose if students taking the module have personal experience, whether it’s themselves or family or friends, you’d have to be very sensitive in the way that it’s delivered*.” (PK)

### Theme 3: Creating a safe space.

Due to the sensitive nature of the topic of suicide, and the potential vulnerabilities of those attending the training, focus group participants recommended that the creation of a safe space was vital for supported, effective learning. The sub-themes that emerged from this theme were (i) early establishment to enable engagement, and (ii) suitable approaches to create safety.

#### (i) Early establishment to enable engagement.

Suicide has proven to be a sensitive topic, therefore the participants felt that it was imperative for the module to create a “*safe space*” from the outset.An environment where students would feel comfortable to be open while talking about the difficult topics of suicide, in order to to support student engagement with the module was recommended.

*“You need to create a safe environment and you’re highlighting the importance of it straight off the bat because I just know that people aren’t necessarily as comfortable talking about it, especially if they’ve either had trauma themselves or they’ve kind of experienced someone close to them having the trauma, they probably would need to be made a little bit more safe and comfortable.”* (PG)

#### (ii) Suitable approaches to create safety.

Participants suggested some ways to create this “*safe space*” early on, from simple methods like sitting in a circle during sessions, to ensuring that group numbers are kept small to be less intimidating. It was thought that these strategies would set up the environment to be as welcoming to students as possible, serving to increase their comfort levels and improve their engagement with the module.

*“Then you can kind of just like bounce off people and stuff. And I think you’re a lot more likely to engage and talk about stuff when there is no kind of power dynamic there.”* (PF)

One participant felt that an important aspect of this “safe space” was for everyone partaking in the module to be seen as equals, including lecturers and students.

*“I’m kind of big into the approach of breaking down that barrier between the lecturers and the students, to create this kind of maybe more relaxed environment.”* (PL)

### Theme 4: Student self-care and support – different strokes for different folks.

Although the main focus of the module under development was suicide prevention training to support patients, the inclusion of a student wellbeing component that was referenced in the module overview was very much welcomed, although the specific content had yet to be finalised. Useful advice and insights from a student perspective were gleaned. The sub-themes that became evident here were (i) need to support students’ own mental health, and (ii) not a “one-size fits all” approach.

#### (i) Need to support students’ own mental health.

Given the stressful environment that often comes with working as a healthcare professional, as well as the recognised risks, participants emphasised the importance of students taking this module to also be supported in looking after their own mental health.

*“I think that this kind of a module is extraordinarily useful for us as students, just because anybody in a clinical setting is going into such a heavy stress profession that we’re the ones that also tend to burn out quite easily”*. (PD)

#### (ii) Not a “one size fits all” approach.

Suggestions were given regarding what type of self-care education to include for students. Participants explained the need for clear instructions when carrying out these activities, to make it easier for students to engage with them. They also suggested providing a choice of evidence-based techniques to avoid the frustration of navigating the myriad of resources online, rather than focusing on one method of self-care, which some people may not enjoy.

*“There’s so many resources, it gets confusing. But the point being that you know, different strokes for different folks and all that so.”* (PA)

*“Students should select self-care like options, that are custom-made for them to specifically address their concerns”.* (PI)

Another important point that was raised was the need to make self-care activities relatable and realistic for students to be able to perform, so as to not set themselves up for failure and therefore reduce their engagement with it. Participants felt that the self-care activities should be easy to complete, initially “*starting out small*” and “*slowly building it up*” (PG), with “*bite size tips*” which can be “*introduced gradually throughout the module, for people to work on them in their own time and to reflect upon them*” (PL). They expressed the importance of this in terms of not overwhelming students initially which would make them say “*that’s not for me*” and then “*you never go back to it again*” (PA).

### Theme 5: Module implementation.

The focus groups explored in great detail the potential ways that this module could be implemented, and discussed the benefits of different approaches, as well as the potential obstacles or challenges that may be faced. The important sub-themes and delivery considerations that emerged from the findings within this theme were (i) compulsory versus elective module, and (ii) online versus in-person delivery.

#### (i) Compulsory vs. elective module.

Overall, the participants held a positive view of the proposed module content and felt that it was quite comprehensive in the broad range of topics it covered.

*“I think the topics looked really good, honestly. I like how you’re kind of covering suicide from, like, a broad point of view. So, like talking about epidemiology and like risk factors and stuff like that, and also going into the view of different health care providers, talking about effective communication etc. So, I thought it looked really good”* (PB).

As one participant stated,

*“I would have loved to have had this in my first year, I would have loved to learn all of this before even entering a rotation because you never know what type of patient will present with what”.* (PI).

This positivity for the module content proposed led to the majority of participants advocating for it to be made compulsory in their respective courses.

Participants noted that the engagement level of students could depend largely on whether or not the module is compulsory, asstudents would have to be more engaged with the module if it was mandatory.

*“If this module was a compulsory module, I don’t think you’d have the option to lose students’ attention. You know, if this was compulsory as part of the degree, they would have to show up and they would have to do it, whereas if it was like an elective that people could drop at any time, I could understand (lack of engagement) a bit more.”* (PG)

It was also discussed that having the module as an elective would mean that it would reach less students as a result, and potentially defeat the purpose of the training, by not equipping all future graduates equally.

Many participants felt that the importance of suicide prevention justified the module being mandatory for healthcare students.

*“I guess everyone in their personal life will have some interaction with this, if it’s not an immediate family member or friend, then it’s only a friend of a friend. I think for that reason it I think it should be mandatory.”* (PA)

When the timing of the module was discussed, it was noted that the sooner it was offered to students, the better. Participants expressed sentiments that they would like to avail of this type of training before they went onto clinical placement, so that they would be adequately prepared before meeting patients.

*“I just think it’s important for it to be before people actually start seeing patients. Because especially if it’s your first time dealing with somebody that’s having suicidal ideation, that can be really harrowing. And so, if it’s not happening before that, then I, I don’t know if it’ll have as much of a use and an impact.”* (PD)

#### (ii) Online vs. in-person delivery.

Participants also debated over what they felt was the best way to deliver this module, whether online or in-person. Initially, participants tended to agree on in-person delivery, because of the sensitive nature of the topic of suicide prevention, as well as the interactive nature of the activities and case studies. This would enable a more open, flowing engagement and richer learning as a result.

*“being behind your computer and turning off your camera… I might just say, just do it all in person, just cause otherwise you don’t have people opening up as much and you won’t have those conversations that you need”*. (PD)

However, some participants favoured the idea of blended learning and suggested that it may *“…work best for everyone because like you’ll get those like people in class then who won’t speak up in person…and may prefer to join in on an online session”*. (PF)

### Theme 6: Appropriate teaching methodologies.

This theme emerged as an important consideration for the module delivery, encompassing consideration of teaching pedagogies, use of specific types of content, and interdisciplinary learning. The key sub-themes here were (i) a move away from the traditional didactic approach, (ii) the power of lived experience, and (iii) interprofessional learning and collaboration.

#### (i) Moving away from the traditional didactic approach.

Many of the participants expressed their preference for practical, engaging teaching methods for this module, rather than traditional didactic lectures, and noted the value of students having to partake in role-play scenarios to further their communication skills in this area.

Participants also advocated for the use of observing role play demonstrations, as it can help students to appreciate specific guidelines on how to act in situations, coming from current healthcare professionals.

*“If there was just a demonstration of an interaction, like “This is an example of someone that’s come into the ED. This is how the intern goes up to them and approaches them” sort of thing. I think that would be really good then, because you kind of just get a feel for what you actually have to say and do.”* (PF)

However, perspectives differed between medical and pharmacy students regarding the feasibility of using role play in teaching related to suicide prevention, with medical students seeming more comfortable with this approach than pharmacy students.

*“I know it would be easy to just do the role plays, like, we have had role plays where someone comes in with the pain in their stomach or whatever. That’s one thing, but I don’t know. Can you do that when it’s this topic, you know it might be disrespectful.”* (PK)

#### (iii) The power of lived experience.

As well as practical scenarios for teaching, the value of having people with lived experience and healthcare professionals to share their experience with suicide or suicide prevention training was recognised across the groups.

*“I think within the classroom, like hearing from doctors or nurses or pharmacists or anyone who’s been in these situations would be helpful. I don’t think that that’s the topic necessarily that you can open the textbook and read and just kind of get it”* (PB).

It was acknowledged that although listening to and seeing such content would be challenging for some, and emotive for most, the value that would come from such learning would be significant. The humanisation of the topic was where participants felt they would benefit the most.

*“I think it wouldn’t be easy to listen to, but it would be the best, most effective way to learn. You can’t argue with it. It’s there, and they’re telling their story. So, while it could be quite challenging, I think it would hammer home the points in the most effective way. It would force you to feel things and realise that it is not just made-up scenarios, that it’s someone’s real life. I just think that definitely adds that reality to it that you might not get if you weren’t doing those lived experiences.”* (PK)

#### (iv) Interprofessional learning and collaboration.

Participants also considered the benefits and drawbacks that could come from this module being run as an interdisciplinary offering, with different healthcare courses being taught together. The positive view of having an interdisciplinary module was the learning that could be gained from different perspectives, teaching the value of multidisciplinary teamwork and building interprofessional collaborations in this area.

*“I think that this could be a really unique opportunity for classes to merge… there’s never the opportunity to do that. So, I think that that would be kind of interesting and just like to meet other people first of all, and then also just to encourage discussion on and seeing how they do intertwine.”* (PB)

*“Everyone’s singing off the same hymn sheet…every profession recognises that they have a role to play rather than it being like a segmented approach.”* (PJ)

Any negative views towards interdisciplinary teaching came from potential logistical considerations.

*“To add to the logistics of how you would implement interdisciplinary courses together. I mean, I don’t know how many people there are in that group. I mean, I think in medicine we have about 200. So, if you have another 300 people, then you have to think about it”* (PC)

A counter argument also emerged for making the module discipline-specific, so that the role for each healthcare course was really emphasised and made clear to students.

*“But then kind of looking at it from the other side, maybe it would be beneficial to really look at it for, medical students specifically, maybe it would be beneficial to really like hammer in exactly how this works in your field and make it more specific.”* (PB)

## Discussion

This study explores the perspectives of undergraduate health and social care students on suicide prevention training and offers valuable insights into how such training could be embedded effectively within Irish third-level curricula, and in a broader context. Across all focus groups, a clear and consistent finding was that students felt under prepared to support individuals at risk of suicide, and strongly believed that more comprehensive training should be a mandatory part of their degree programmes. This echoes the findings of international literature, indicating gaps in mental health and suicide prevention education for healthcare students and professionals, as well as the need for earlier, structured intervention [20,21,6,9–12,22,23]. In the Irish context, there is currently no standardised approach to suicide prevention education across undergraduate health and social care programmes. While many higher education institutions do incorporate some aspects of mental health or suicide-related content, delivery is inconsistent and often dependent on individual educators or available resources.

One of the key challenges in embedding suicide prevention training more systematically is the already overcrowded nature of healthcare curricula. Students are required to develop a broad range of clinical competencies in a limited timeframe, and suicide prevention education must often compete with other essential clinical training priorities. As a result, many students may graduate without receiving sufficient preparation to recognise or respond appropriately to suicide risk. This study directly addresses this gap by incorporating student voices into the design of a national, standardised suicide prevention module. By gathering perspectives from those currently enrolled in health and social care programmes, this work ensures that the training is not only evidence-informed but also relevant, practical, and responsive to the educational realities faced by healthcare students in Ireland. It represents a significant step toward developing a scalable and sustainable model for integrating suicide prevention education across the Irish healthcare education landscape.

Participants attributed the current lack of suicide prevention training to several interrelated factors, including institutional fears of triggering distress in the students, lack of knowledge of the topic, and/or a lack of competency-focused indication for such training. Despite these barriers, participants in this study overwhelmingly supported the inclusion of such training and offered insights into how it could be delivered in a feasible and supportive manner. Notably, students expressed a strong preference for mandatory over elective training, reinforcing that suicide prevention should be considered a core competency for all healthcare graduates. There was also consensus that training should include in-person, interactive elements that foster engagement, confidence, and emotional preparedness—echoing findings from LeCloux (2021) [24] which gathered Master of Social Work students’ (n = 45) opinions on the delivery of online asynchronous suicide prevention training. Preferences here were variable, with the majority of students (44.7%) indicating that they would prefer to receive content related to suicide in-person, a quarter (25.5%) preferring a blended format, 4.3% preferring online delivery only, and 25.5% indicating no preference at all [24].

Importantly, participants also emphasised the need to move away from the traditional didactic lecture approach and incorporate more experiential learning strategies—particularly teaching through lived experience. There was an appreciation from participants that this module was different from their usual science-based courses, and that there would be a need to adapt a different set of techniques to enhance the learning experience. This is clearly supported in the literature, which indicates that teaching through lived experience enables students to gain a greater understanding of their own attitudes, self-awareness, and fears, as well as an appreciation for the impact of mental illness on an individual level – qualities that are essential in any healthcare role [25–28].

Multiple participants expressed the sentiment that as future healthcare professionals, they need to be well equipped to deal with suicidal patients, as they will encounter and care for them in their future careers. This need for comprehensive training is further underscored by findings from a recent mixed-methods survey of community pharmacists in Ireland, which revealed that 61% of respondents (n = 134/219) had experienced the death of a patient by suicide, yet 88.5% had received no formal suicide prevention training [29]. Free-text responses highlighted significant uncertainty around how to respond to patients at risk, with many pharmacists reporting they did not know what to say or how to act in these situations—largely due to a lack of training or prior guidance [29]. These findings closely mirror those of the present study, further illustrating the pressing need for structured suicide prevention education across all healthcare disciplines.

While participants acknowledged the potential emotional challenges associated with suicide prevention training—particularly for students with previous experiences of trauma—they consistently emphasised that the benefits of such training far outweighed the risk of distress. As a means of minimising any potential distress, participants emphasised the importance of personal self-care and wellbeing education being delivered throughout. Interestingly, a recent scoping review (*n* = 147 articles) investigating the extent of self-care education among medical students concluded that psycho-emotional support and self-care education is under-recognised, underdeveloped, and poorly integrated in medical student’s curricula [30]. These findings highlight the need to embed self-care as a core component of suicide prevention training, ensuring that students are provided with a variety of options for self-care techniques that are student-centred. Additionally, providing students with the resources and support to access appropriate mental health and wellbeing services is essential.

The potential for interprofessional learning was a striking theme highlighted by students. Suicide prevention is a public health issue and involves multidisciplinary contributions across primary and secondary care. Learning with, from and of one another in any context as a healthcare professional is recognised as being a useful way to bridge gaps in care in practice [31]. A recent study of interprofessional collaboration by healthcare students by Fleming et al (2024) reported that following a group exercise of working through a clinical patient case, student feedback was overwhelmingly positive including positive agreement in all four AITCS-II sub-scales (partnership, coordination, cooperation and teamworking [32]. Clear benefits in developing clinical knowledge and attitudes towards collaborative practice and teamworking were evident, an approach that this module in suicide prevention could draw upon.^29^ Suicide prevention specific examples of interprofessional learning are also provided in the literature. Gorton et al (2021) evaluated the learning of pharmacist and mental health nursing students via a pre and post session survey. A statistically significant increase in preparedness was reported in both pharmacy students (p < 0.005) and MHN students (p < 0.005), following the session (n = 76 in paired analysis) [33]. This is a promising indication for future work that brings multiple healthcare courses together.

These insights have important implications for both policy and curriculum design.

Importantly, the module discussed in this study directly aligns with *Connecting for Life: Ireland’s National Strategy to Reduce Suicide*, specifically Goal 5: “Ensure the provision of high-quality services” [16]. However, broader systemic change is required to ensure that suicide prevention education is not viewed as optional or peripheral. Engagement with professional regulatory bodies such as CORU, the Nursing and Midwifery Board of Ireland, and the Irish Medical Council will be essential to advocate for the inclusion of suicide prevention within existing competency frameworks. Doing so would provide academics and programme leaders with a policy-backed rationale to support curricular inclusion and help ensure consistency across institutions.

Institutional feasibility is another key consideration for delivery of such training. While some barriers exist—Including staff expertise, timetabling constraints, and the need for psychological safety protocols—there are also promising signs of practical feasibility. Many institutions already deliver mental health education or interprofessional learning, both of which offer natural entry points for suicide prevention training. Moreover, blended or modular delivery formats can help ease the burden on academic timetables, while partnerships with external organisations (e.g., mental health charities, lived experience networks) could support content delivery.

Finally, cost considerations should not be overlooked. While there are upfront resource needs associated with developing and delivering a new module—such as staff time, training materials, and facilitator support—these must be weighed against the broader public health and professional benefits. Training that equips future health professionals to intervene early in suicidal crises may reduce longer-term costs associated with emergency care, repeated presentations, and adverse outcomes for patients and families [34]. Furthermore, the potential for interprofessional delivery offers efficiencies by allowing shared sessions across multiple programmes.

### Limitations

While this work has provided valuable insights into students’ opinions and needs for suicide prevention training in their undergraduate programmes, it is not without its limitations. Unfortunately, representation was not achieved of students from all undergraduate health or social care programmes, due to the restricted recruitment time-frame, despite efforts made to capture the viewpoints of other courses. This may make this work less generalisable. The convenience sampling strategy employed may have introduced some bias and reduced external validity. However, due to the time constraints of the project, it was decided that convenience sampling would be the most feasible option for this piece of work, and all efforts to ensure data comprehensiveness and transferability were undertaken.

Another limitation relates to the self-reported nature of the data, which may be subject to social desirability bias. Participants may have offered responses that they believed were expected or appropriate, particularly given the sensitivity of the topic. Furthermore, the voluntary nature of participation may have excluded input from students who felt uncomfortable discussing suicide-related content, despite the presence of appropriate ethical and psychological safety protocols.

Lastly, while this study captured student perspectives at a single point in time, attitudes and experiences may evolve throughout their education and future clinical practice. Longitudinal research would be beneficial in understanding how needs and perceptions around suicide prevention training shift over time, and how such training ultimately impacts professional competence and confidence in the field.

## Conclusions

Despite the limitations outlined above, the data generated by this study have provided rich insights and invaluable guidance for the piloting of this module and for educators internationally. The findings have illustrated the evident desire of healthcare students to receive suicide prevention training as part of their undergraduate education. This will help to ensure that the needs and requirements of those taking this module are met, ultimately improving patient and suicide prevention outcomes in clinical practice. The personal wellbeing of the healthcare student is also recognised as an integral component of the module, inextricably linked to patient care, with potential for interprofessional learning throughout. The results of this study have been used to inform further iterations of the module, and its subsequent pilot and evaluation. This is an important step towards standardised suicide prevention training for all health and social care students, seeking to optimise patient care in this challenging area.

## Supporting information

S1 File Module Content Information for Participants. (DOCX)

S2 File Focus Group Topic Guide. (DOCX)
