# Supplementary material for: Understanding the needs of undergraduate healthcare students in relation to suicide prevention training: A qualitative study
Source: PLoS One. 2025 Jul 9;20(7):e0327538. doi: 10.1371/journal.pone.0327538 (PMC12240323; doi:10.1371/journal.pone.0327538)
Supplement: S1 File — (DOCX) [file pone.0327538.s001.docx]

**Supplemental File 1: Module content information for participants**

**Development of a suicide prevention module for undergraduate health and social care students**

Important information for focus group participants

**“Understanding the needs of students in relation to suicide prevention training”.**

Dear Participant,

Thank you for your interest and participation in this study.

This study aims to explore the perspectives of students enrolled in health and social care courses regarding the development of a suicide prevention module. The purpose of this document is to summarise the module’s content development to date, which will be a central component of the discussion on the day. It will be presented briefly at the outset of the focus group, but it would also be helpful for you to familiarise yourself with it prior to the session, if possible.

| Module Topic | Content included | Potential Learning Activities |
| --- | --- | --- |
| Pre-module session | - Creating a safe environment - Trigger warnings for content - Importance of self-care and wellbeing - Importance of suicide prevention training for healthcare students/professionals | - Attitude Polls - Discussion on student expectations for module - The voice of lived experience. - Self-care activity – 7 days of self-care |
| Topic 1:  Epidemiology of Suicide / Suicide as a Public Health Problem | - Definitions of suicide and self-harm - Epidemiology of suicide - Attitudes and myths concerning suicide | - Creating a shared digital library for resources, e.g., self-care resources, readings, mental health and suicide prevention resources - Finish session with a self-care activity |
| Topic 2:  Risk and Protective Factors | - Individual, relationship, community and societal risk and protective factors - The social determinants of suicide - Suicide prevention for specific groups | - Group brainstorm activity - Written assignment on specific population at increased risk for suicide - Finish with a self-care activity |
| Topic 3:  Preventing Suicide and Self-harm | - Public health approaches to preventing suicide/self-harm - Universal, selective and indicated approaches to prevention - Media portrayal of suicide and self-harm - Ireland’s evidence-based prevention strategies | - Group exercise – What is the role of your profession in suicide prevention. Give 1 to 2 examples of each level on intervention and who it may target? - Group exercise – look at media articles that report on suicide, what do you notice? Are there differences in reporting? - Finish with self-care activity |
| Topic 4:  Effective Communication | - Barriers that prevent people from seeking help - The parameters of confidentiality - Direct and indirect warning signs - A professional approach to communication - Signposting to local resources and support services | - Case study / role play - The voice of lived experience. - Finish with a self-care activity |
| Topic 5:  Risk Assessment, Safety Planning and Prevention in Clinical Practice | - Clinical management of self-harm - Responding to individuals presenting with self-harm and suicidal ideation in clinical practice - Risk assessment and formulation - Components of care in the emergency department (ED) - Safety planning - Module conclusion and reflection on learning | - Group exercise - brainstorm what might help or not help before and during a administering a biopsychosocial assessment. - Role play - Case study – identify the role of different health and social care professionals in a patients journey from ED to aftercare – potential interprofessional learning session. - Finish with a self-care activity |

We look forward to discussing the development of this module with you and gaining insights into the student perspective on its content and delivery.

Many thanks in advance for your time and participation.

Yours sincerely,

The research team
