# Supplementary material for: Understanding the needs of undergraduate healthcare students in relation to suicide prevention training: A qualitative study
Source: PLoS One. 2025 Jul 9;20(7):e0327538. doi: 10.1371/journal.pone.0327538 (PMC12240323; doi:10.1371/journal.pone.0327538)
Supplement: S2 File — (DOCX) [file pone.0327538.s002.docx]

**Supplemental File 2: Focus Group Topic Guide**

**Focus Group Interview Questions**

**Study title: Understanding the needs of students in relation to suicide prevention training.**

*Opening address*

- Confidentiality.
- Focus group etiquette.
- Reminder that this is voluntary, and they are free to withdraw.
- Purpose for teaching and learning not personal experience.

| Section | Questions |
| --- | --- |
| Part 1 (5 mins)  Introductory questions  “To start off...” | 1. Would you mind briefly introducing yourself and telling us your name, course and any previous experience you have from your undergraduate teaching/training on the topic of mental health or suicide prevention? |
| Part 2 (15 mins)  Lecture content  “Now I would like to move on to discuss your opinions on the content of the proposed module” | 1. What are your thoughts on the content and the topics proposed to be included in this module? Any additional suggestions from anyone else?    1. How relevant are they to training of health and social care students for suicide prevention?    2. If you were taking this module, would you be happy with the level of content delivered to you?      1. Are there any aspects outside the suggested curriculum that you think we have missed or that you think would be beneficial to be included in the module, but which have not been mentioned? 2. What are your opinions on including a self-care component to this module and what would that look like? 3. Can you think of any additional resources that you might need to be provided? 4. Given the nature of this topic, can you think of any difficulties that may arise in its delivery?   *Prompt: For example, stigma around suicide, difficulty of approaching the subject and talking about it outright with a patient who has suicidal ideation?* |
| Part 3 (20 mins)  Your needs as a student  (How) | 1. What do you think is the most successful way to deliver this module to students? For example, do you think it should be run as a blended learning (with both online and in person components) or should it be completely run in person?   *Prompt : for example the use of role plays, or do you have any other suggestions?*   1. What teaching methods do you think would best support your ability to learn and improve your skills in dealing with patients with suicidal ideation? 2. What are your thoughts on the usefulness of having examples of lived experience in the teaching of this module, for example inclusion of interviews with someone who has experienced suicidal ideation or who has been bereaved by suicide? 3. How do you feel about the proposed structure of a week on with a two-hour class and a week off for reflection and self-directed learning? 4. What teaching methods do you think would best support your ability to learn and improve your skills in dealing with patients with suicidal ideation? 5. How do you think this module should be graded or assessed? 6. How do you think this module should be included in the year’s teaching curriculum? For example, should it be its own stand-alone 5 credit module, or should it be part of another module, or should it be made as an elective?    1. What do you think the benefits and drawbacks of all these suggestions would be? 7. Should this be made as a stand-alone module for each course, or should it be taught as an interdisciplinary module with other healthcare courses?    1. Have you had any experience with interdisciplinary teaching modules?    2. If so, what was your experience with it?    3. What were the benefits?    4. Could you see these benefits applying to this module if it was held as an interdisciplinary module? |
| Part 4 (5 mins)  Future Recommendations | 1. Before we leave this is there any other ideas you have that could improve the module programme? Or do you have any other extra recommendations? 2. Do you have any further comments or inputs that you would like to make before we finish up? |
